# A nutrition intervention with a main focus on vegetables and bread consumption among young men in the Norwegian National Guard

**DOI:** 10.3402/fnr.v57i0.21036

**Published:** 2013-10-21

**Authors:** Solveig Uglem, Tonje Holte Stea, Marte Karoline Råberg Kjøllesdal, Wenche Frølich, Margareta Wandel

**Affiliations:** 1 Department of Nutrition, Institute of Basic Medical Sciences, University of Oslo, Norway; 2 Department of Public Health, Sport and Nutrition, University of Agder, Norway; 3 Norwegian School of Hotel Management, Department of Business Administration, University of Stavanger, Norway

**Keywords:** intervention, young men, vegetables, bread

## Abstract

**Background:**

Young men are difficult to reach with conventional nutrition information and they have a low intake of vegetables and whole grain cereals. Few intervention studies have focused on improving young men's consumption of vegetables and whole grains.

**Objective:**

A 5-month intervention focusing on a combination of increased availability of healthy foods and nutritional information was developed to stimulate the intake of vegetables and semi-whole grain bread among a group of young men in the Norwegian military.

**Subjects:**

A total of 376 recruits in the intervention group and 105 recruits in the control group participated in the entire study.

**Results:**

The average daily increase in consumption of vegetables was 82 g (*p*<0.001), and semi-whole grain bread 47 g (*p*<0.001) between baseline and follow-up in the intervention group. No significant changes were observed in the control group. Differences between intervention and control group at follow-up were significant (*p*<0.001) for vegetables and semi-whole grain bread, when controlling for baseline values, and seasonal variation for vegetables. The recruits in the intervention group received higher scores on the questions concerning nutritional knowledge after the intervention, compared to baseline (*p*<0.001). There was a significantly higher increase in the intake of vegetables among the recruits who increased the number of correct answers to the knowledge questions (*β*-value: 0.14, *p*<0.05) than among the others. There was no significant change in scores of food satisfaction after the intervention.

**Conclusion:**

The combination of increased availability of healthy food items and nutrition information was an effective way to increase the intake of vegetables and semi-whole grain bread, without a reduction in food satisfaction, among young men in the military.

Young men are difficult to reach with conventional nutrition information. Studies have shown that men often are less interested in nutrition and health than women, and that the middle aged and older men often are more interested than the younger ones, who tend to take health for granted (1–3). There is a need for increased knowledge about successful means to improve the diet among men, especially those who are not very interested in nutrition and health.

Plant-based foods, such as vegetables, fruits, and whole grain cereals are given increased attention, since they may protect against obesity and related chronic diseases (4–6). Both Norwegian and other European studies have shown that the intake of vegetables and whole grains is lower than what is recommended (7–9). In Norway, only 44% of men aged 18–29 years reported a daily consumption of vegetables (10), and only 27% of young men reported a consumption of whole grains in line with the official recommendations (8, 10).

Norwegian military service is compulsory for young men, and they are supposed to be randomly distributed to different military camps in Norway. Thus, military camps are unique settings to reach young men of different socio-economic, cultural, and geographical backgrounds. In these institutional settings, the possibilities for making own decisions regarding food choice might be somewhat limited (11). However, dietary studies among Norwegian recruits have shown large variations regarding the intake of healthy foods, such as fruits and vegetables, and whole grain bread (12), indicating that the recruits have some opportunities to exert their own choice even in the military setting.

There is limited research on interventions targeting eating habits in the military (11). Most of these studies are from the United States. They include nutrition education (13, 14), modification of food services (15), nutrition labeling to facilitate informed food choices (16), and behavioral change through multiple component programs, with focus on a supportive environment for weight loss (17–19). However, the recruitment to military service in the United States is different to the recruitment in many European countries, making the results less comparable.

Scandinavian intervention studies in military settings have shown varying results. An intervention study carried out at a garrison canteen in Denmark, focusing on increased availability of fruits and vegetables, resulted in increased consumption of these foods, and long-term sustainability (20, 21). A Finnish multicomponent intervention study showed positive effects on consumption frequency of porridges and cereals, fatty foods, and sugar-rich foods, such as soft drinks and desserts, but failed to increase the consumption of fruits and vegetables among the recruits (11). Variation in results may be explained by different factors, both related to study participants and to the military environment. Thus, there is a need to identify more detailed characteristics of the study participants and settings of intervention studies in order to further develop tailored intervention studies focusing on this important target group.

The main aim of this study was to investigate if the combination of nutrition information and increased availability of vegetables and semi-whole grain bread led to an increased consumption of these foods in a group of young men in the military. The study also investigated differences in consumption of fruit, juice, and potatoes, as well as nutrition knowledge, and food satisfaction before and after the intervention.

## Method

### Study design and subjects

**Fig. 1:**
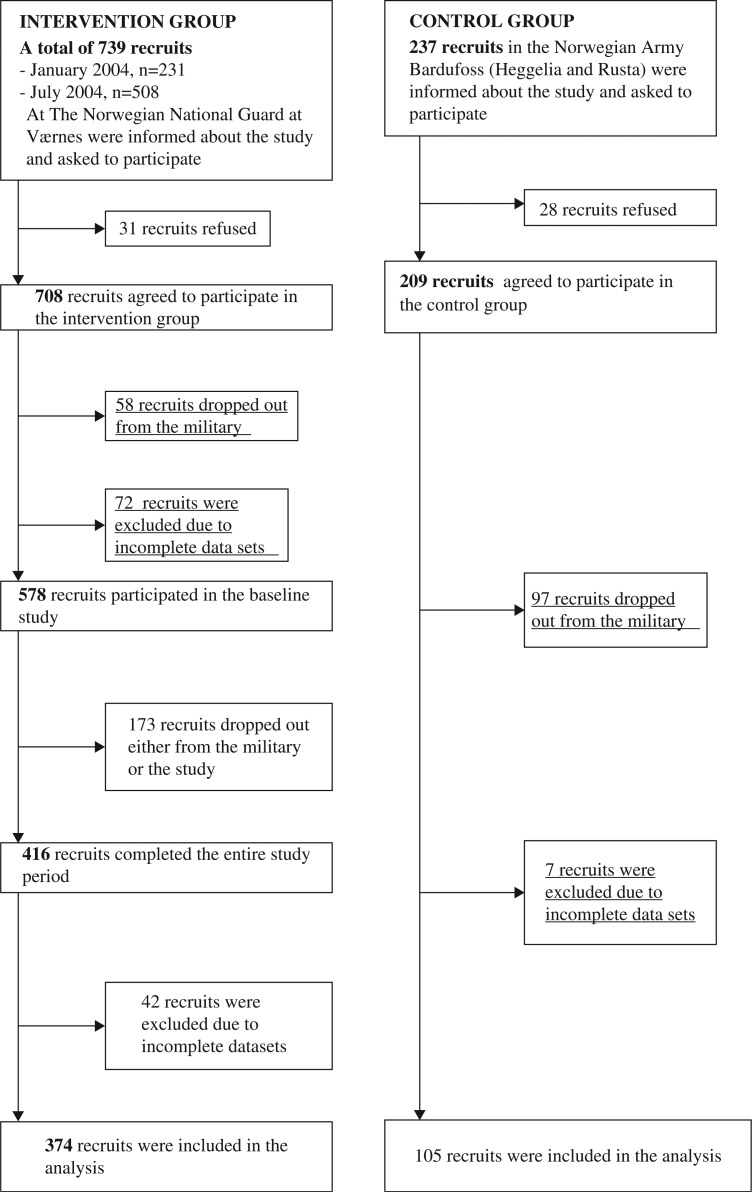
Flow chart of the participants.

All recruits who followed ordinary military service in specific enrolments in two military camps in Norway were asked to participate in the study. The recruits in Vaernes Military Training Centre close to Trondheim were assigned as the intervention group and recruits in Bardufoss military training centre as the control group (Figure 1). A total of 376 recruits in the intervention group and 105 recruits in the control group participated in the entire study. The same intervention was performed with two enrolments at Vaernes military training centre; the first from January to May 2004 and the second from July to November 2004. Data were collected with a diary and a questionnaire right before the intervention (baseline) and right after the 5-month intervention (follow-up). For the control group, the same data were collected at baseline in September 2005 and at follow-up in February 2006. All participants were male. Data collection was carried out by two of the authors (S. U. and T. H. S.) who lived in the camps for extended periods during data collection and intervention. Research assistants participated in some parts of the data collection. The project also benefitted from collaboration with the military personnel. Ethical approval and research clearance was obtained from The Norwegian Social Science Data Services, The Ministry of Health and Care Services, and The Regional Committee for Medical Research Ethics.

### Intervention program

The intervention was designed based on a model of factors affecting food choice, presented in another publication (12). The intervention was directed at the kitchen personnel to change the food environment, especially the availability of vegetables and semi-whole grain bread, as well as to the recruits, with nutrition information related to vegetables, fruits, and whole grain cereals. The intervention consisted of the following components:

#### Increased availability of vegetables and semi-whole grain bread

The availability of healthy food items were increased in several ways:

1. A self-service salad bar consisting of a large variety of vegetables was introduced for the lunch meal.
2. For dinner, vegetables were included in newly developed dishes, or vegetables were offered as side dishes.
3. Bread with a wholegrain content of 50–100%, and a fiber content of 4–7 g/100 g was offered to all meals.

New recipes with a large amount of vegetables were developed by one of the researchers (T. H. S.) in cooperation with professional chefs, and the nutritional content of the dishes was calculated. Prior to the intervention the cooks at Vaernes Military Training Centre were trained by the professional chefs in preparing the new dishes, and in presenting additional vegetables in appealing ways.

#### Nutritional education in the eating environment

Information about the health benefits of a diet rich in vegetables, fruits, and whole grain cereals, as well as more detailed information about the different foods that were in focus in the study, were given to the recruits in an information meeting and through posters, brochures and folders. The posters were placed at strategic places in the mess hall where the recruits could study them. Three different posters, five of each, were present at the same time, being replaced with new versions every 6 weeks. The posters contained information about main health effects of vegetables and whole grain bread. The posters also gave information about the Norwegian recommendations of fruit and vegetables, ‘5-a-day’, and about bioactive compounds.

Brochures and folders from several Norwegian health organizations and agricultural boards were placed in the mess hall, available for the recruits to read. Furthermore, two project workers were available for 2 days about every sixth week to answer questions from the recruits, in addition to their staying in the camps. The recruits could also write questions and comments about the study and put these in a mailbox in the mess hall. However, only a few of the recruits put questions and comments in the mailbox. More recruits questioned the researchers directly during data collection and intervention periods.

### Dietary assessment methods

Two survey instruments were developed: a diary to assess the participants’ intake of vegetables, potatoes, bread, fruit, and juice, and a questionnaire to assess nutritional knowledge of the recruits, and their satisfaction of the food offered in the military mess hall. The participants completed the diary in 4 working days for each survey period. The questionnaire was completed on the first day of each survey period. Both the diary and the questionnaire were tested in a pilot study with 12 recruits in a military camp in Norway (Lutvann) and were revised on the basis of the results and comments from this group. The revised survey instruments were test–retested with fairly acceptable responses over a 3-week period among 63 recruits in another military camp in Norway (Heistadmoen) (12).

#### Food diary

A validated diary developed at the Department of Nutrition, University of Oslo (http://www.helsedirektoratet.no/publikasjoner/ungkost-2000-landsomfattende-kostholdsundersokelse-blant-elever-i-4-og-8klasse-i-norge/Sider/default.aspx) was modified to reflect the diet in the mess hall. The diary gave pre-printed choices of frequency and size for estimation of the participants’ consumption of the selected food items. For hot dishes the diary had open-ended questions where the participants filled in a number for the day's special. The diary also included questions about how often and what they ate in the canteen at the camp and/or cafeterias/restaurants outside the military camp.

When appropriate, the participants stated their consumption as pieces of vegetables. For hot dishes served at lunch and dinner and some other items, like salad, the participants stated their intake as number of servings and serving sizes (small, medium, large, or extra-large). Serving sizes for these food items were decided with guidance from the kitchen personnel and from other Norwegian surveys (22). To decide their own serving sizes, the subjects were asked to refer to photographs or weighed portion sizes placed on a table in the mess room.

To obtain estimates of the participants’ consumption of vegetables in grams, pieces of most vegetables printed in the diary, were pre-weighed. The estimates are the average of 10 weighed pieces. For some items, like potatoes, standard measures in Norway were used (23). To collect data of the participants’ consumption of vegetables included in the dishes, recipes and estimates of the amounts of the ingredients used in every dish were given by the kitchen personnel. Average contents of vegetables in the different serving sizes were calculated from these data. The total intake of vegetables (in g/d) was calculated as the product of frequency of intake and serving size.

The intake of bread was measured as slices of bread. The recruits had three pre-printed choices of bread; white bread, semi-whole grain bread and whole grain bread. However, most of the bread offered in the mess hall was semi-whole grain, and thus the recorded intake of whole grain bread was low. We observed that some of the recruits had mistaken semi-whole grain bread for whole grain, because it was colored with malted flour and had a dark color. Thus, we used the sum of semi-whole grain and whole grain bread in the analyses, and called it semi-whole grain bread.

#### Knowledge

Four questions were developed to assess the recruit's knowledge about the nutritional value of plant-based food, with pre-printed scales with one correct answer, three wrong answers and don't know. The questions were: (i) In what kind of food do you think there is the most antioxidants? (answers: meat, fish, vegetables, cereals or do not know); (ii) Which food do you think contains most fiber per weight? (answers: meat, fish, vegetables, cereals or do not know); (iii) What do you think is the name of the bioactive substance in fruit and vegetables which may prevent cancer? (answers: carcinogens, antioxidants, enzymes, lipoproteins or do not know); (iv) How many grams of fruits and vegetables do you think is recommended to be eaten daily by an adult? (answers: 250 g, 500 g, 750 g, 1,000 g, do not know). The participants were given 1 if the answers were correct and 0 if they were wrong or did not know.

#### Food satisfaction

The degree of satisfaction with the food offered in the military mess hall was measured by the question: ‘How satisfied are you with the food in the military mess hall’. This was measured on a 5-point scale: very satisfied, rather satisfied, neither satisfied nor unsatisfied, not really satisfied, not satisfied at all. This was made into a dichotomous variable for analysis; very satisfied and rather satisfied were coded as satisfaction, and the other three response categories as not satisfaction.

### Analyses

Data were analyzed using SPSS 18.0. Chi-square tests and independent sample *t*-tests were used to investigate differences between groups at baseline. Changes in intake between baseline and follow-up were analyzed using related sample *t*-tests. Differences in intake at follow-up, adjusted for baseline intake were analyzed using ANCOVA. Since the intake of vegetables, fruits, and potatoes is prone to seasonal variations, adjustments for seasonal variation were made for these foods. Only those who had reported intake at ≥3 days were included in the analyses. Changes in knowledge and satisfaction were analysed with Chi-square and McNemar tests. Linear regression analyses were carried out to explore associations between increased nutritional knowledge of plant-based foods and changes in intake of vegetables and of whole grain bread.

## Results

Table 1 presents age characteristics of the participants, and their socio-economic background, indicated by their mother's and father's educational level. Survey respondents were aged between 18 and 26 years, with an average age of 19.7 years in the intervention group and 19.2 years in the control group. There was a significant difference in father's educational level between the intervention and the control group (*p*<0.05), but no significant difference in mother's educational level. Most of the recruits in both groups had completed high school.

**Table 1:** Descriptives of the participants at baseline

	Intervention	Control
Age (mean, SD)	19.7 (2.8)	19.2 (1.3)*
Mother's education (%)		
Primary school	13.1	13.9
High school	37.0	40.5
University/college ≤3 år	20.9	22.8
University/college ≥4 år	28.9	22.8
Father's education (%)		
Primary school	13.1	13.0
High school	38.6	38.5
University/college ≤3 år	12.0	19.9**
University/college ≥4 år	36.3	28.6**

* p<0.05 (t-test).

** p<0.05 (chi-square test).

The intake of vegetables at baseline varied from 0 to >500 g/d, with an average of 143 g/d in the intervention group and 107 g/d in the control group (Table 2). Nearly 63% of the participants in the intervention group, and nearly 80% in the control group, consumed <150 g/d of vegetables at baseline. The combined intake of fruit and fruit juice was 281 g/d in the intervention group and 349 g/d in the control group, with juice contributing to the major part.

**Table 2:** Average intake of selected food items in the intervention and control group at baseline and follow-up

Food items		Average intake (g/d) baseline	SE	Average intake (g/d) follow-up (adjusted for baseline intake*)	SE	Within group** *p*-value	Between groups at follow-up*** *p*-value
Vegetables	Intervention	143	4.6	222 (225)	5.5	<0.001	<0.001
	Control	107	9.2	99 (88)	11.2	0.45	
Fruit	Intervention	128	5.8	144 (142)	5.6	0.012	<0.001
	Control	111	14.2	83 (90)	11.3	0.040	
Juice	Intervention	153	9.5	178 (183)	9.2	0.119	0.001
	Control	238	19.7	280 (255)	18.6	0.024	
Potatoes	Intervention	101	3	46 (47)	2.6	<0.001	<0.001
	Control	130	7	104 (101)	5.2	<0.001	
White bread	Intervention	14	1.3	19 (20)	2.1	0.02	0.23
	Control	20	4.1	26 (25)	17	0.26	
Semi-whole grain bread	Intervention	160	3.8	206 (207)	4.5	<0.001	<0.001
	Control	164	7.7	152 (151)	9.6	0.216	
Eating <150 g vegetables/d (%)	Intervention	62.8		33.8		<0.001	
	Control	77.9		75.8		1.00	

Only participants having answered the questions 3 or 4 days at both baseline and follow-up.

* For vegetables, fruit, juice, and potatoes (and salad) also adjusted for season at follow-up (winter/summer).

** Related sample *t*-test.

*** ANCOVA, adjusted for baseline values (for vegetables, fruit, juice, and potatoes also adjusted for season at follow-up [winter/summer]).

*N* =374–376 (intervention) and 84–99 (control).

Significant increases in consumption of vegetables, fruit, and semi-whole grain bread were demonstrated between baseline and follow-up in the intervention group (Table 2). The increase in vegetable intake, adjusted for baseline in the intervention group was 82 g/d. No significant change in vegetable intake was observed in the control group. During the course of the intervention, the frequency of recruits who consumed <150 g vegetables was reduced from 63 to nearly 34% (*p*<0.001) in the intervention group. No significant reduction was observed in the control group. The difference in vegetable intake between the intervention and control group at follow-up was 137 g/d, adjusted for baseline and seasonal variation (*p*<0.001).

A significant reduction in the consumption of potatoes of 55 g/d (*p*<0.001) in the intervention group was observed, and the intake of fruits increased on average 14 g/d in the intervention group (*p*<0.05). In the control group, a significant reduction between the baseline and follow-up in the consumption of both fruit (*p*<0.05) and potatoes (*p*<0.001) was observed (Table 2). The difference in fruit intake between the intervention and control group at follow-up was 52 g/d, adjusted for baseline and seasonal variation (*p*<0.001). The intake of potatoes in the intervention group was half of that in the control group at follow-up.

The intake of semi-whole grain bread increased on average 47 g/d (*p*<0.001) in the intervention group. No significant change was observed in the control group. There was a significantly higher intake of semi-whole grain bread in the intervention group compared with the control group at follow-up (56 g/d, *p*<0.001).

The recruits in the intervention group scored higher on the questions about nutritional knowledge of plant-based foods after the intervention, compared to baseline (Table 3). At baseline, 30% of the recruits did not give any correct answers to the knowledge questions, while at follow-up this number was reduced to nearly half. The percentage of recruits who had three out of four correct answers were nearly four-doubled over baseline levels (*p*<0.001). The results from linear regression analysis showed a significantly higher increase in the intake of vegetables among the recruits who increased the number of correct answers by at least one (*β*-value: 0.14, *p*<0.05) than among the others.

**Table 3:** Percentages with correct answers on knowledge of nutritional value of fruits, vegetables and whole grain cereals

		Baseline	Follow-up	Change within group, p*	Difference in change between groups, p*
Intervention	0 correct	29.8	14.6	<0.001	<0.001
	1 correct	45.7	24.0		
	2 correct	16.4	27.5		
	3 correct	6.3	24.7		
	4 correct	1.8	9.1		
Control	0 correct	33.8	33.8	1.000	
	1 correct	59.7	59.7		
	2 correct	6.5	6.5		
	3 correct	0	0		
	4 correct	0	0		

* Chi-square tests (for composite index, difference in proportions having increased number of correct answers with at least 1).

*N* (only those with answer both at baseline and follow-up): intervention: 396–403, control: 139–144.

The changes for each number of correct answers were all significant in the intervention but not in the control group (McNemar test).

There was a small and insignificant increase (1.2%) in the number of recruits in the intervention group who reported to be satisfied (very satisfied and rather satisfied) with the food offered in the military mess hall after the intervention. The changes in satisfaction were not significantly different between the intervention and control group.

## Discussion

This study showed that a combination of increased availability and nutritional information may help to increase young men's intake of vegetables and semi-whole grain bread toward what has been recommended by official authorities, without a reduction in the food satisfaction. The intervention also had a small but significant positive effect on the intake of fruit and fruit juice, whereas the intake of potatoes was reduced.

Most of the earlier intervention studies targeting fruits and vegetables have used combined measures of fruits and vegetables. This study treated vegetables, fruits, juice, and potatoes as separate entities, both in the intervention strategies and in data collection and analyses. This choice was made, since these foods generally have different roles in a diet. Furthermore, these foods may have differential effects in relation to various diseases, and are therefore not interchangeable in nutrition recommendations (8).

The Norwegian recommendations state that the daily intake of vegetables and fruits/berries should be at least 500 g/d, half of which should be vegetables and the other half fruits/berries (8). Fruit juice can be included in the fruit/berry part, limited to one glass. Thus, the average intake of vegetables of 143 g/d at baseline in the intervention group is far below the recommendations. This low intake is in agreement with other studies among young adults (7, 24–26). The collected intake of fruit and fruit juice at baseline in the present study met the recommendations, even when the addition of fruit juice was limited to 1 glass/d.

An increase in intake of vegetables of 82 g/d, semi-whole grain bread of 47 g/d and fruits of 14 g/d was observed among the recruits in the intervention group. The main aim of the intervention was to increase the recruit's intake of vegetables and whole grain bread, and therefore there was less focus on fruits. Thus, it is interesting to note that the intake of vegetables and bread increased more than the intake of fruits. Interventions focusing on multiple strategies have shown promising results also in other population groups, particularly when the strategies have included increased availability of the desired food items (20, 27).

It is difficult to compare the effect of the present intervention with other studies because of the combined measure of fruit and vegetables in most other studies. Furthermore, the intake was measured in gram per day in the present study while many other studies use daily servings. The size of a recommended serving may also vary, as the United States recommendations use 80 g/serving, whereas most European recommendations use 100 g (8, 28). In the Danish study, which focused on the availability in the canteen of the military base, the fruit and vegetable intake increased by 70 g right after the intervention, and by 78 g after 1-year follow-up (20, 21). How much of this increase that is due to the vegetable consumption is not specified, but it can be assumed that it is <78 g. The study by Gambera (13) of US air force members getting nutrition counselling, showed an increase of 2.5 servings of fruit, but no significant change in the intake of vegetables. Thus, the increase in vegetable consumption in the present study is higher than in these other studies. The increased intake of high-fiber bread is in agreement with an intervention study among obese men, who showed an increased intake during and 20 months after the intervention (29).

The intervention group in this study increased their nutritional knowledge substantially and the results showed association between increased knowledge and increased intake of vegetables. The results show that posters, brochures, and folders with simple and focused nutritional messages available in the eating environment were effective. In agreement with the findings from studies on other population groups, they indicate that increased knowledge may have implications for consumption of fruits and vegetables (30–33), when nutrition information is part of a multiple component program.

A strength of the study is the compulsory military service practiced in Norway, which made it possible to reach a wide variety of participants in terms of socio-economic status, geographical origin, food habits, and interest in food and health (12). A strength regarding the intake data is the detailed measurements of vegetables and fruits, and that the recipes of the composite dishes were known so that the vegetable content could be calculated rather precisely. It is a limitation that the recruits did not get the information to be able to record precise information on the whole grain content of the bread, and that misunderstandings occurred regarding semi-whole grain and whole grain bread. It is also a limitation that the recordings of the food eaten outside the military mess hall were not of the same good quality as the food eaten in the mess hall.

A limitation affecting the generalizability of the study is that the young men had some restrictions in their food choices. However, for both lunch and dinner they had the choice of a hot dish or bread with spreads. There were also always offers of side dishes of vegetables, salad from a salad bar, and fruits. In addition, they had the possibility to buy food in a commercial canteen or in restaurants or grocery stores outside the camp. Thus, they had many choices which resulted in a large variation in intake of vegetables and fruits (12).

Another limitation, affecting the strength of the study, is related to the fact that the control group included few participants. Unfortunately, changes in the enrolment in the military made it impossible to obtain equal groups. The differences between the intervention and control group regarding socio-economic status (father's educational level) is also a limitation. Furthermore, since the follow-up period is short, we do not know the long-term effects of the intervention.

In conclusion, this study showed that a combination of increased availability and nutritional information increased the intake of vegetables of about 80 g/d, and semi-whole grain of nearly 50 g, in a group of young men in the military, without reduction of their satisfaction with the food. The positive results from this intervention study indicate that it is possible to get young men to undertake dietary changes. These results could be an inspiration for other interventions among young men. More research is needed among young men in other settings, for example school canteens and other work places with young men. Research is also needed to determine the long-term effects of dietary interventions among young men.
